# From silenced shock to strategic resilience: a longitudinal qualitative study of nurse residents’ trajectory in coping with patient verbal abuse

**DOI:** 10.3389/fpsyg.2026.1780789

**Published:** 2026-06-19

**Authors:** Hui Luo, Kejimu Sunzi, Xia Lv, Yu Liao, Jie Huang

**Affiliations:** 1 School of Intelligent Nursing, Sichuan Nursing Vocational College, Chengdu, Sichuan, China; 2 People’s Hospital of Deyang City, Deyang, Sichuan, China; 3 The First Affiliated Hospital of Chengdu Medical College, Chengdu, Sichuan, China

**Keywords:** cognitive appraisal, coping trajectory, longitudinal qualitative research, newly graduated nurses, nurse residents, patient verbal abuse, workplace violence

## Abstract

**Background:**

In China, newly graduated nurses are required to complete a standardized 1-year clinical residency, a transition marked by high stress and limited authority. Patient verbal abuse is common and often normalized as “part of the job,” yet it can destabilize emotions and shape early career trajectories. Longitudinal evidence on how nurse residents interpret and cope with patient verbal abuse across their first year remains limited. Grounded in Lazarus and Folkman’s transactional theory, this study examines trajectories of appraisal and coping in response to patient verbal abuse during the first year of practice.

**Methods:**

We conducted a longitudinal qualitative study in a high-volume tertiary hospital in Southwest China. Eighteen nurse residents were recruited through maximum-variation purposive sampling and completed three waves of semi-structured interviews at approximately 1, 6, and 12 months of residency (T1–T3).

**Results:**

Four themes captured the evolving trajectory: (1) initial emotional shock and vulnerability; (2) negotiation of hierarchical and cultural constraints; (3) gradual acquisition of adaptive strategies; and (4) tension between professional maturation and gaps in institutional support. Over time, primary appraisals often shifted from threat-focused interpretations toward more differentiated threat–challenge evaluations. Coping moved from early avoidance and suppression to more proactive, skill-based responses, while reporting remained selective.

**Conclusion:**

Verbal abuse operates as a dynamic stressor embedded in the transition to practice rather than a one-off incident. The findings support a phase-matched approach to residency support, progressing from early psychological validation to de-escalation skills training and system-level actions that rebuild trust in reporting pathways.

## 1 Introduction

Globally, the healthcare sector continues to grapple with workplace violence (WPV) as a critical occupational hazard rather than a sporadic problem (Tang et al., 2025; Jakobsson et al., 2020). Evidence suggests that WPV affecting health professionals is most often non-physical, with verbal abuse from patients or visitors being particularly common. Nurses may be disproportionately exposed because of their prolonged, high-intensity contact with service users (Bedin-Zanatta et al., 2021). Recent studies also indicate that patient verbal abuse toward nursing staff is reported more frequently than physical violence, posing a persistent threat to workforce well-being and retention (Toska et al., 2023).

Historically, clinical culture has tacitly normalized behaviors such as yelling, insults, and derogatory threats as “part of the job” (Lall et al., 2021). This normalization is increasingly untenable. Accumulating evidence links patient verbal abuse to emotional exhaustion, reduced job engagement, and higher turnover intention (İlhan et al., 2025). The perceived futility of reporting may contribute to persistent underreporting, which in turn can limit organizational learning and delay timely intervention (Aljohani et al., 2021; Sethi et al., 2024).

In China, WPV has gained increasing research and policy attention. Meta-analytic evidence indicates a high annual incidence of WPV among hospital nurses, with verbal violence accounting for a large proportion (Lu et al., 2020). These patterns have been discussed in relation to structural pressures such as staffing shortages, overcrowding, and prolonged wait times, alongside strained patient–provider relationships (Goldschmitt et al., 2025; Williams et al., 2022).

Nurse residents–new graduates navigating China’s standardized 1-year clinical training–occupy a particularly precarious position. Compared with transition arrangements in which trainee and workforce roles are more clearly separated, Chinese nurse residents often hold a hybrid identity as both “learners” and essential “workforce” (Yao et al., 2023). This liminal status may limit perceived protection and authority, potentially increasing vulnerability to patient-initiated aggression. While global literature acknowledges that novices frequently encounter aggression during the transition to practice (Alshawush et al., 2022), research in China remains largely cross-sectional. Moreover, verbal abuse is often subsumed under broad categories of psychological violence, obscuring its specific dynamics. As a result, theory-guided longitudinal insight into how nurse residents appraise and cope with verbal abuse across their first year of practice remains limited.

To address these gaps, we adopted a longitudinal qualitative design grounded in Lazarus and Folkman’s transactional model of stress and coping. Lazarus and Folkman’s transactional theory conceptualizes stress as a dynamic relationship between individuals and their environment rather than as a property of the event alone. In this model, primary appraisal refers to how individuals evaluate whether an encounter is irrelevant, benign, threatening, harmful, or challenging, whereas secondary appraisal concerns their perceived resources and options for managing the situation. Coping may involve emotion-focused strategies, such as avoidance, suppression, or emotional regulation, and problem-focused strategies, such as seeking support, reporting, boundary-setting, or using communication skills. This framework was therefore well suited to examining how nurse residents’ interpretations of patient verbal abuse and their coping resources changed across the first year of practice. We followed Chinese nurse residents at three time points during their first year to examine trajectories of responses to patient verbal abuse. Specifically, we aimed (1) to explore how residents interpret and appraise abusive incidents; (2) to identify changes in emotional reactions, help-seeking behaviors, and trajectories of appraisal and coping over time; and (3) to analyze individual, interpersonal, and organizational factors shaping adaptation and professional socialization. Ultimately, the findings are intended to inform stage-specific interventions, ranging from communication training to system-level reforms, to support a safer transition for nurse residents.

## 2 Materials and methods

### 2.1 Design and theoretical framework

We used a longitudinal qualitative design to examine how Chinese nurse residents interpret and cope with patient verbal abuse during their first-year transition to practice. This approach enabled us to capture changes over time, including continuity and shifts in meaning-making, appraisal, and coping. Guided by Lazarus and Folkman’s transactional model of stress and coping, we traced changes in participants’ primary and secondary appraisals, emotional responses, coping strategies, and help-seeking across the residency year (Lazarus and Folkman, 1984).

### 2.2 Setting

Fieldwork was conducted at Deyang People’s Hospital, a high-volume tertiary hospital in Southwest China. The hospital’s fast-paced workflow and high patient throughput provided a context in which nurse residents may encounter patient-initiated patient verbal abuse during early clinical practice (Yuan et al., 2025).

### 2.3 Participants and sampling

We defined nurse residents as newly graduated nurses enrolled in the standardized clinical training program required during their first year of practice (Luo et al., 2025). To capture a broad range of early-career experiences, we used maximum-variation purposive sampling across gender, educational background, and clinical placement contexts. During recruitment, the research team used a sampling matrix to monitor variation in sex, degree level, and clinical units or rotation areas. This strategy was intended to include residents with different exposure contexts, patient-contact patterns, and supervisory environments within the same standardized residency program.

Variation in clinical placement contexts was monitored across interview waves, including medical wards, surgical wards, emergency department, intensive care unit, obstetrics/gynecology, pediatrics, and other units; these contextual characteristics are reported in Supplementary Table 2.

Participants were eligible if they (1) were enrolled in the standardized residency program, (2) were within their first year of practice, and (3) were willing to participate in three interview waves. We excluded residents on extended leave or unlikely to be available for follow-up. Sampling continued until additional interviews yielded no substantively new insights into appraisal and coping trajectories across waves, and the longitudinal dataset was judged to provide sufficient depth and variation.

### 2.4 Data collection

#### 2.4.1 Interview timeline and procedures

The cohort comprised 18 nurse residents who completed three rounds of semi-structured interviews at 1 month (T1), 6 months (T2), and 12 months (T3). These time points were chosen to capture key stages of the transition to practice: early adjustment (T1), mid-year adaptation (T2), and later-stage consolidation (T3). We achieved zero attrition across waves. To ensure voluntariness, participants were informed that participation was independent of training evaluation and that they could withdraw at any time without consequences; consent was re-confirmed at each wave.

Interviews were conducted face-to-face in private teaching rooms within the hospital, with no supervisors present. Data collection was led by researchers who had no supervisory or evaluative authority over the residents to minimize power differentials and facilitate candid disclosure. Sessions typically lasted 35–45 min, were audio-recorded with consent, and conducted in Mandarin. Field notes were taken to capture contextual details and non-verbal cues.

#### 2.4.2 Interview guide development

The initial interview guide was informed by WPV literature and the stress-and-coping framework, focusing on concrete abuse incidents, appraisal processes, emotional responses, coping actions, and perceived professional impact (Kafle et al., 2022). The guide was iteratively refined across waves; insights from T1 informed additional probes for T2 and T3. For example, after “professional masking” emerged at T1, we added targeted questions in the T2 guide to explore the emotional costs and perceived consequences of suppression strategies (Supplementary Table 1). The main interview questions and wave-specific probes are summarized in Supplementary Table 1, which presents the T1, T2, and T3 interview focus areas and sample questions derived from the stress-and-coping framework. Participants were not asked to complete a formal resilience self-assessment or resilience scale; however, the interviews included open-ended probes concerning their perceived ability to recover from abusive encounters, confidence in handling similar incidents, perceived changes in coping resources, and reflections on whether these experiences contributed to professional growth.

#### 2.4.3 Translation of interview materials and quotations

Interviews were transcribed verbatim in Mandarin Chinese and de-identified prior to analysis. For publication, illustrative quotations were translated into English by a bilingual nurse researcher. A second bilingual researcher independently checked translations against the original transcripts to ensure semantic and pragmatic equivalence (including tone, implied meaning, and context-specific expressions). Discrepancies were resolved through discussion and, when necessary, by consulting the audio recordings. Culturally specific terms (e.g., Chi ku, “endurance”) were retained in pinyin at first mention and briefly explained to preserve meaning.

### 2.5 Data analysis

Given the longitudinal design and the larger sample size (*N* = 18) relative to traditional idiographic IPA studies, we combined interpretative phenomenological analysis (IPA) with a longitudinal case-based matrix approach to retain within-person depth while enabling systematic temporal comparison. Each participant was treated as a case. We first developed time-ordered case summaries (T1–T3) to trace shifts in primary and secondary appraisal, emotional responses, coping actions, and help-seeking decisions before moving to cross-case synthesis. To retain IPA’s idiographic commitment, we produced a coherent longitudinal narrative for each participant prior to any cross-case comparison and avoided frequency-based counting as the primary basis for interpretation.

Analytic procedures proceeded in four steps. (1) Within each time point, transcripts were read and re-read and annotated with exploratory comments (descriptive, linguistic, and conceptual), followed by the generation of emergent themes. (2) For each participant, emergent themes from T1, T2, and T3 were assembled into an individual longitudinal matrix (case × time) to identify continuities, turning points, and mechanisms of change. (3) We then compared cases to identify convergent and divergent patterns and to build higher-order interpretative themes. To avoid losing idiographic detail, we repeatedly returned to each participant’s case matrix and supporting quotations during synthesis. (4) We conducted negative case analysis by intentionally identifying trajectories that did not fit the dominant pattern (e.g., sustained threat-focused appraisals or avoidant coping) and used these cases to refine theme boundaries and prevent overgeneralization of a single developmental pathway.

The first author led the initial coding and memo writing. Two additional researchers independently reviewed a subset (approximately one-third) of transcripts, sampled across T1–T3, together with accompanying analytic notes. Divergent interpretations were resolved through discussion and by returning to the transcripts and, when needed, the audio recordings. To strengthen dependability, we maintained a detailed audit trail documenting analytic decisions from raw quotations to emergent themes and longitudinal placement. Turning points were defined as sustained shifts in appraisal or coping across waves, supported by explicit within-case narrative contrasts and exemplar quotations in the case matrix. A worked example of the analytic chain (quotation → exploratory notes → emergent theme → longitudinal placement) is provided in Supplementary Table 4. NVivo was used to organize transcripts, memos, and matrices; interpretative decisions were made through iterative team discussion rather than automated coding.

Lazarus and Folkman’s transactional theory informed interpretation as a sensitizing framework rather than a fixed coding template. We remained open to inductive meaning-making and mapped findings onto appraisal and coping constructs only after themes had been developed to minimize theory-driven overfitting.

### 2.6 Rigor and trustworthiness

To enhance rigor, we used multiple strategies (Ahmed, 2024). Credibility was supported through iterative team debriefings and cross-wave comparisons. The absence of supervisory relationships between researchers and participants helped reduce social desirability and encouraged candid accounts. Dependability was strengthened through the audit trail and the independent review of a subset of transcripts and analytic notes by additional researchers. Confirmability was supported through reflexive memos documenting analytic decisions, assumptions, and alternative interpretations. Transferability was facilitated by detailed description of the residency context and setting.

### 2.7 Research team and reflexivity

The research team comprised nurse educators and researchers trained in qualitative inquiry. Interviewers had no supervisory or evaluative role in the residency program. Reflexivity was maintained through regular meetings and written memos that documented assumptions, emotional responses to the data, and alternative interpretative possibilities. Throughout analysis, we discussed how our clinical and educational backgrounds might shape interpretations and used reflexive notes to bracket preconceptions when developing and refining themes.

### 2.8 Ethical considerations

The study received ethical approval from the Ethics Committee of Deyang People’s Hospital (Approval No. 2023-04-081-K01). Written informed consent was obtained prior to the first interview and re-confirmed at subsequent waves. Participants were reminded of their right to withdraw at any time without consequences. Transcripts were de-identified and participants were assigned codes (P1–P18). All digital data were stored on password-protected devices accessible only to the research team.

### 2.9 Reporting standards

This manuscript follows the COREQ (Consolidated Criteria for Reporting Qualitative Research) checklist to promote transparency in study design, analysis, and reporting (Dossett et al., 2021).

## 3 Results

### 3.1 Participant characteristics

The study retained all 18 nurse residents across three interview waves, yielding a complete longitudinal dataset. Participants were predominantly young and female: 77.8% were women, and 61.1% were aged 20–22 years (Table 1). Over half held an associate degree (55.6%) (Table 1). Contextual exposure changed across the year; night-shift exposure increased over time (77.8% at T1, 88.9% at T2, and 94.4% at T3) (Supplementary Table 2). Notably, none had received any structured WPV-related training (e.g., de-escalation or reporting training) prior to T1.

**TABLE 1:** Baseline demographic characteristics of nurse residents at T1 (*N* = 18).

Characteristics	Category	*n* (%)
Gender	Male	4 (22.2)
Gender	Female	14 (77.8)
Age (years)	20–22	11 (61.1)
Age (years)	23–25	7 (38.9)
Education	Associate degree	10 (55.6)
Education	Bachelor’s degree	8 (44.4)

Demographic variables (age, sex, education) were collected at T1 and were unchanged across waves. Percentages are based on *N* = 18.

### 3.2 Overview of longitudinal findings

Analysis yielded four themes describing residents’ trajectories: (1) initial emotional shock and psychological vulnerability; (2) negotiation of hierarchical and cultural constraints; (3) gradual development of adaptive coping strategies; and (4) tension between professional maturation and gaps in institutional support. Across waves, appraisals shifted from threat-oriented interpretations marked by definitional ambiguity and normalization at T1 toward more differentiated threat–challenge evaluations by T3. In parallel, coping evolved from avoidance and suppression to more proactive, skill-based responses, although reporting remained selective and contingent on incident severity (Table 2).

**TABLE 2:** Longitudinal evolution of themes and coping mechanisms across the first year of practice.

Themes	T1 (1 month): entry phase	T2 (6 months): adaptation phase	T3 (12 months): stabilization phase
Theme 1: Emotional shock and vulnerability	Shock and ambiguity: Unable to distinguish abuse from venting; self-doubt; normalizing abuse to survive.	Lingering impact: Acute shock fades, but subtle micro-aggressions continue to erode confidence; rumination.	Cognitive reframing: Differentiation between “pain-induced” and “personal” attacks; “threat” turns to “challenge.”
Theme 2: Navigation of constraints	Professional masking: Surface acting; fear of “troublemaker” label; suppressing authentic emotions.	Hidden exhaustion: Masking causes internal strain; reliance on peers rather than supervisors.	Role stabilization: Supportive mentorship validates experience; feeling safe to drop the “mask.”
Theme 3: Coping strategies	Avoidance: Crying with peers; silence; passive endurance.	Testing boundaries: Attempting explanatory communication; venting in private groups.	Integrated coping: Skill-based de-escalation; selective reporting; focus on task completion.
Theme 4: Institutional support	Gap in training: Felt unprepared; unclear reporting pathways.	Variable response: Help-seeking depends on luck (supportive vs. dismissive leaders).	Demand for system: Desire for formal training and transparent protection protocols.

#### 3.2.1 Negative case trajectories: persistent threat-focused appraisal

Although the dominant trajectory involved gradual movement toward more differentiated appraisal and integrated coping, negative case analysis identified a small number of residents whose adaptation remained more constrained. Across waves, these participants continued to interpret patient verbal abuse primarily as a sign of personal inadequacy, occupational threat, or lack of protection. Their coping remained largely avoidant or suppressive, and they were less likely to use formal reporting pathways unless the incident was severe or witnessed by others. These cases suggested that adaptation was not a linear or universal process and that the absence of supervisory validation or credible reporting feedback could maintain threat-focused appraisal over time. Detailed exemplar quotations for each subtheme across time points are provided in the Supplementary Table 3.

P6, T3: “Even now, when a patient scolds me, my first reaction is still to wonder whether I did something wrong. I know I have worked for almost a year, but I still cannot completely separate their words from my own ability.”

P14, T3: “I rarely report these incidents, not because they no longer affect me, but because I do not think reporting will change anything. Sometimes I also worry that leaders will think I am too sensitive or troublesome.”

### 3.3 Theme 1: emotional shock and psychological vulnerability

#### 3.3.1 Definitional ambiguity and early normalization (T1)

Upon entry into practice, residents often described uncertainty about whether a patient’s outburst constituted verbal abuse or illness-related venting. This ambiguity was frequently accompanied by self-doubt and hesitation to label the incident as “abuse,” which contributed to non-disclosure. One participant described blaming herself rather than the aggressor and choosing not to report the incident:

P1, T1: “When the family member pointed at me and said, ‘How can you not know how to do anything?’, my first thought was, ‘Is it because I really didn’t do well enough or was too slow?’ I kept wondering whether this counted as being bullied or whether he was just too anxious. In the end I didn’t tell anyone. I felt maybe this is just what work is like, and speaking up would make me look like I can’t handle pressure.”

Residents also described a perceived expectation to empathize with patients, which sometimes conflicted with their own distress and further discouraged formal reporting:

P12, T1: “The patient yelled some nasty words at me. I froze there. Later my preceptor asked what was wrong, and I said I was fine. But inside I felt terrible. I also thought the patient was in pain, so maybe that was why. If I went to a supervisor, it might look like I was being petty and couldn’t endure hardship.”

#### 3.3.2 First-encounter shock and emotional unpreparedness (T1)

Residents described early encounters as emotionally jarring, often contrasting them with the empathic nurse–patient relationship emphasized in nursing education. For some, these initial experiences were accompanied by immediate doubt about career fit:

P5, T1: “I was truly stunned. At school, teachers talk about empathy and caring. I was completely unprepared for someone to curse you with such harsh words. That whole day I felt distracted and unsettled, and I wondered whether I had chosen the wrong profession.”

A similar sense of early career doubt was expressed by another participant, who linked the first abusive encounter to a sudden loss of confidence in her readiness for nursing work:

P7, T1: “I had imagined that patients might be dissatisfied, but I did not expect someone to speak to me with such harsh words. At that moment, I felt embarrassed and helpless, and I kept asking myself whether I was too inexperienced or too fragile. After work, I still could not calm down and wondered whether I was really ready to become a nurse.”

#### 3.3.3 Lingering impact of subtle remarks (T1–T2)

Beyond overt outbursts, residents reported that subtle sarcasm or dismissive remarks could have a lingering impact, contributing to heightened self-monitoring and performance anxiety. One resident described how a single remark persisted and shaped subsequent clinical confidence:

P3, T1: “That grandfather looked me up and down and said to his daughter, ‘Why did they send a little rookie? Can she even figure it out?’ He didn’t say it loudly, but I heard every word. That sentence kept circling in my head. After that, I became very nervous before every procedure, constantly wanting to prove myself.”

By mid-year, some residents described being able to maintain outward composure while still experiencing prolonged internal effects:

P8, T2: “Now when I hear those sarcastic remarks like, ‘Oh, another new one today?’, I can smile it off on the outside. But inside it sticks for a long time. I keep thinking whether I did something that made me seem unprofessional.”

#### 3.3.4 Emerging regulation and reframing (T3)

By the 12-months interview, several residents described a growing ability to differentiate pain-driven outbursts from personal attacks, which helped them depersonalize the incident and regulate emotions:

P17, T3: “Now I can more or less tell the difference. If someone lashes out because of pain or fear, I try to understand and not take it as personal. I know this is not my failure but that they are going through suffering. Of course, pure personal attacks are a different matter.”

Another participant described a similar reframing process, emphasizing that increased clinical exposure helped her interpret some verbal outbursts as expressions of fear, pain, or uncertainty rather than as direct personal rejection:

P15, T3: “Now I try to judge the situation first. If the patient is in obvious pain or fear, I remind myself that the anger may not be aimed at me personally. This helps me stay calmer and continue the care. But if the words become insulting or threatening, I know it is no longer something I should simply endure.”

### 3.4 Theme 2: struggling within hierarchical and cultural constraints

#### 3.4.1 Professional masking and emotional suppression (T1)

At T1, residents described maintaining a calm and compliant outward demeanor during abusive encounters while suppressing their emotional reactions. This “masking” was often linked to concerns about escalating the situation and being judged as unable to cope:

P2, T1: “I kept a smile on my face, saying, ‘Please don’t be angry, I understand’, but my hands were shaking and my stomach was twisting. I absolutely couldn’t cry or talk back, otherwise the situation would get messier, and the teachers would think you can’t handle things.”

#### 3.4.2 Hierarchical constraints and fear of negative labeling (T1–T2)

Residents reported that hierarchical relationships within the ward shaped whether and how they sought help. At T1, many described reluctance to report incidents because they feared being perceived as “troublesome” or as having poor communication skills:

P9, T1: “I didn’t dare tell the head nurse. She might think I provoked the family because of my communication, or that I was making a big deal out of it and causing trouble for the unit. What nurse residents fear most is being labeled as ‘too much trouble’.”

By T2, some residents described prior dismissive responses from senior staff, which reinforced a preference for sharing experiences with peers rather than approaching preceptors or supervisors:

P14, T2: “Once I mentioned it to an experienced teacher. She sighed and said, ‘If you can’t even take this, what will you do later?’ After that, I was more inclined to vent with my peers from the same cohort–at least they understand you.”

#### 3.4.3 Stabilization via mentorship and experience (T3)

In contrast, residents also described how supportive mentorship could change how they interpreted and responded to verbal abuse. By T3, some participants emphasized that validation from a preceptor and practical guidance contributed to a stronger sense of belonging and confidence:

P11, T3: “My preceptor later shared similar experiences from when she was young and taught me a few ways to respond. More importantly, she made me feel it wasn’t my fault. With her backing me up, I no longer felt like a lonely rookie.”

Another resident similarly emphasized that mentorship helped transform the incident from a personal failure into a manageable workplace problem:

P10, T3: “My preceptor helped me review the situation instead of simply telling me to endure it. She said the patient’s anger was not necessarily my fault and taught me how to explain, pause, and call for help if needed. After that, I felt more secure in the ward.”

### 3.5 Theme 3: gradual development of coping and adaptive strategies

#### 3.5.1 Informal peer support as the safest early resource (T1–T2)

During the early transition (T1), residents frequently described relying on peers for emotional validation and reassurance. Sharing experiences with cohort members helped reduce feelings of isolation and self-blame:

P4, T1: “After work I cried with my roommate (also a new nurse). We comforted each other, saying, ‘It’s not our fault.’ The feeling of ‘so you’ve been yelled at too’ immediately made me feel less lonely and less ashamed.”

By T2, some residents described more organized and private peer support structures, such as group chats without supervisors, which functioned as a confidential space to vent and exchange coping ideas:

P16, T2: “We have a small group chat with no teachers in it. It’s become our ‘tree hole’ (a private venting space). Whoever gets upset or tries a comeback shares it there. We vent, give each other ideas, and stick together to get through it.”

#### 3.5.2 Expansion into skill-based coping and de-escalation (T2)

At T2, residents described beginning to test more proactive, skill-based responses in interactions with patients and families. One commonly reported approach was expectation-setting in advance of procedures:

P7, T2: “Now I proactively explain first: ‘Auntie Wang, this injection may hurt a bit. I’ll try to be gentle, but I may need you to stay still.’ When I say it in advance, they actually complain less. I learned this from a teacher, and it really works.”

#### 3.5.3 Integrated coping and selective institutional engagement (T3)

By T3, residents described combining emotional regulation with task-focused communication and more selective engagement with formal reporting pathways. Many indicated they were more likely to document or seek support when verbal abuse felt persistent, overtly threatening, or likely to affect care, and when some form of corroboration (e.g., a witness or written record) was available. In contrast, residents often reported handling single, illness-related outbursts privately through de-escalation and self-regulation. Concerns about being negatively labeled, the time burden of reporting, and limited feedback from the organization were frequently cited as reasons for non-reporting.

P15, T3: “Now I force myself to calm down first. I don’t follow their bait. I just say, ‘Let’s deal with your problem first’, and bring the focus back to treatment. Afterward I calm myself down. But if what they say is really too much or feels threatening, I’ll note it in the handover book or mention it privately to a leader I trust. I know it may not always help, but what should be documented must be documented, and what should be said still needs to be said.”

### 3.6 Theme 4: tension between professional growth and institutional support

#### 3.6.1 Early threats to professional identity (T1)

At T1, residents described verbal abuse as undermining confidence in their professional competence and triggering self-doubt about career fit:

P6, T1: “After being pointed at and yelled at ‘Get out’, I hid in the treatment room with my mind completely blank. The only thought I had was: maybe I really can’t do this job. I felt utterly useless.”

#### 3.6.2 Uneven supervisory responses shaping help-seeking (T2)

At T2, residents described how supervisory responses shaped whether they sought help and how they made sense of the incident. When supervisors validated the resident’s experience and offered guidance, participants reported feeling supported and learning practical ways to respond:

P10, T2: “My head nurse was wonderful. After I finished, the first thing she said was, ‘You’ve been wronged.’ Then she helped me analyze what happened and told me how I could handle it next time. I felt protected, and I learned something.”

In contrast, some residents described dismissive or minimal responses that discouraged further disclosure:

P13, T2: “I reported it to my preceptor. She just said ‘mm-hmm’ without even looking up and continued working on the chart. It felt like a bucket of cold water. As if I was bothering her with something trivial that I should have handled myself.”

#### 3.6.3 Demand for structured training and clear pathways (T3)

By T3, residents articulated a desire for clearer institutional guidance and practical training related to verbal abuse, particularly regarding how to respond and how reporting processes work:

P18, T3: “The hospital trained us in many procedures and theories, but how to respond to nasty words is entirely up to us to figure out. I really wish there had been clear guidance when we started: who to go to, how to report, and what would happen afterward. This shouldn’t be an ‘unspoken rule’ we have to decode ourselves.”

### 3.7 Longitudinal synthesis

Tracing across the first year, residents described an observable shift in how they understood and responded to patient verbal abuse (Box 1). At T1, accounts commonly reflected definitional uncertainty, strong emotional reactions, and a tendency to maintain outward composure while managing distress privately. By T2, residents more frequently described relying on peer support and beginning to test proactive communication strategies in clinical interactions. By T3, many reported greater emotional regulation and more selective engagement with institutional pathways (e.g., documenting incidents or seeking support in specific circumstances), alongside increased expectations for clear guidance and responsive reporting systems.

BOX 1 **Longitudinal case tracer: the trajectory of participant 5.**

To illustrate within-person change, we present a case tracer of Participant 5 across the three interview waves. T1 (1 month): Participant 5 described feeling shocked and questioning career fit after an early episode of verbal abuse. T2 (6 months): Participant 5 described attempting expectation-setting before procedures and relying on peers for emotional processing when verbal abuse occurred. T3 (12 months): Participant 5 described increased emotional regulation and a more practical orientation toward managing incidents, including selectively documenting or seeking support when verbal abuse felt excessive or threatening. Note. This case tracer summarizes the participant’s trajectory across waves; additional exemplar quotations for each subtheme are provided in Supplementary Table 3.

## 4 Discussion

Anchored in Lazarus and Folkman’s transactional theory, this longitudinal study explores Chinese nurse residents’ appraisal and coping trajectories in response to patient verbal abuse over the first year of practice (Cho et al., 2020). Our findings suggest that verbal abuse is experienced not as a one-off occupational inconvenience but as a dynamic stressor embedded in the transition to professional work. Across three interview waves, residents’ accounts indicated an overall shift from early uncertainty and shock (T1), through a period marked by hierarchical and cultural constraints (T1–T2), toward more integrated coping and clearer expectations for institutional support (T3). This pattern is broadly consistent with transactional theory in that residents’ interpretations appeared to move from predominantly threat-oriented appraisals toward more differentiated threat–challenge perspectives. Importantly, these changes unfolded within an organizational context: supervisory responses and reporting climates appeared to function as contextual conditions that either facilitated or hindered adaptive coping (Balducci et al., 2024). At the same time, negative cases–where threat-focused appraisals persisted–indicate that adaptation was heterogeneous rather than uniform.

### 4.1 A time-sensitive trajectory of appraisal: from ambiguity and threat to differentiated interpretations

At the start of residency (T1), many residents described difficulty distinguishing verbal abuse from stress-related venting. This ambiguity was often accompanied by self-doubt and a tendency to normalize incidents, particularly in the absence of prior workplace-violence (WPV) training that could provide clear interpretive cues. These accounts can be interpreted, within the transactional model, as reflecting threat-oriented primary appraisals and reliance on emotion-focused coping such as avoidance or suppression (Sun et al., 2024). Residents also described how sarcastic or dismissive remarks could linger and destabilize early professional confidence, consistent with evidence that early supervisory and workplace experiences shape identity formation during clinical transition (King et al., 2020).

By T3, many participants reported greater capacity to differentiate pain-driven outbursts from personal attacks and to interpret incidents in a more situational manner. This shift–from seeing abuse as a reflection of personal incompetence to viewing it as a context-dependent stressor–may help preserve self-worth while maintaining engagement with care (Daigneault and Brown, 2023). However, this pattern was not universal; a subset of residents continued to interpret verbal abuse predominantly as a threat, underscoring variability in appraisal trajectories.

### 4.2 Hierarchical and cultural constraints as structural brakes on help-seeking

Transitioning to practice involves navigating both psychological stressors and hierarchical workplace dynamics. During T1–T2, residents frequently described reputational concerns (e.g., being labeled “oversensitive” or “troublesome”) as a barrier to disclosure and help-seeking. In these accounts, “professional masking” appeared as a commonly used response, aligning with the concept of surface acting–suppressing authentic emotion to meet perceived workplace display rules (Carminati, 2021). While such strategies can stabilize interactions in the moment, sustained suppression may contribute to accumulated emotional strain, consistent with longitudinal evidence linking emotional labor to adverse mental health outcomes (Suh and Punnett, 2021).

Culturally, an endurance-oriented norm (Chi ku) appeared salient and was often reinforced by workplace expectations. While perseverance may be protective in some contexts, residents’ narratives suggest it can also discourage help-seeking and normalize silence (Qiu et al., 2024). By framing endurance as a marker of professional maturity, such norms may reinforce organizational silence and weaken reporting behaviors (Wen et al., 2025; Morrison, 2023).

Finally, it is important to consider the structural conditions under which “adaptation” occurs. In a mandatory residency with limited exit options, some coping narratives may reflect constrained adaptation rather than freely chosen strategies. This highlights a practical challenge for nursing management: supporting professional development while avoiding the normalization of unacceptable aggression and strengthening credible, responsive reporting pathways (Young et al., 2023).

### 4.3 Progressive development of coping: from peer refuge to skill-based and integrated strategies

Residents’ coping responses appeared to expand in a staged manner across the year. At T1, many relied primarily on peers for validation and emotional processing. By T2, residents more often described experimenting with proactive communication and de-escalation approaches, which may reflect increased perceived control in patient interactions. By T3, participants commonly reported combining emotion regulation with task-focused communication and more selective documentation or escalation. This overall pattern is broadly consistent with the transactional model, in which accumulating experience and resources can shift coping from predominantly emotion-focused strategies toward more problem-focused responses (Cummerow et al., 2023; Labrague, 2024). Importantly, residents’ accounts suggest these changes were shaped by the responsiveness of the surrounding work environment, rather than occurring solely at the individual level (Edmondson and Bransby, 2023).

### 4.4 Organizational responses as accelerators or brakes of adaptation

A key implication of the findings is that organizational responses may modify how residents adapt over time. Residents’ descriptions of “masking” and non-disclosure often reflected anticipated reputational costs and uncertainty about whether speaking up would be supported. Conversely, when supervisors validated residents’ experiences and provided guidance, residents described greater confidence and clearer behavioral options, which may facilitate learning through observation and experience (Krishna et al., 2024).

Accordingly, selective reporting at T3 should be interpreted cautiously. Rather than indicating reduced need for support, it may reflect pragmatic decisions shaped by perceived effectiveness of formal pathways and expected organizational follow-up. Strengthening trust in reporting mechanisms and ensuring visible, timely responses may therefore be central to institutional prevention and support (Spencer et al., 2023).

### 4.5 Stage-specific implications for residency training and retention

The findings support a phase-matched approach to residency support (Table 3).

**TABLE 3:** Phase-matched support components for nurse residency programs across the first year.

Phase	Key needs identified in this study	Training focus	Mentorship/support strategy	Reporting/system strategy
Entry phase T1: ∼1 month	Definitional ambiguity; emotional shock; self-doubt; professional masking; lack of prior WPV training	Orientation on verbal abuse definitions, acceptable boundaries, emotional first aid, and minimum documentation standards	Brief post-incident check-ins by preceptors or head nurses; validation that abuse is not a sign of personal failure	Clear reporting algorithm: who to contact, what to document, and expected response time
Adaptation phase T2: ∼6 months	Hierarchical constraints; reliance on peers; surface-acting strain; early attempts at communication strategies	Simulation-based de-escalation training, including expectation-setting, boundary statements, and escalation triage	Structured debriefing and guided peer reflection with confidentiality rules; supervisor coaching scripts for non-minimizing responses	Non-punitive reporting culture; feedback on whether and how reported incidents are handled
Consolidation phase T3: ∼12 months	Integrated coping but selective reporting; expectations for fairness; trust gaps in institutional follow-up	Advanced scenario training on repeated verbal abuse, threats, and complex family interactions	Reinforced mentorship focused on role-modeling boundary-setting, documentation, and escalation decisions	Closed-loop reporting system, anti-retaliation protection, transparent follow-up, and routine incident review

WPV, workplace violence.

In addition, elements of this phase-matched model could be introduced before licensure or before entry into residency. Pre-licensure simulation scenarios involving patient verbal abuse may help students rehearse boundary-setting language, de-escalation techniques, emotional regulation, documentation, and reporting decisions in a psychologically safe environment. Such simulation-based preparation may reduce the gap between classroom ideals of empathic care and the realities of emotionally charged clinical encounters.

Entry phase (T1): Programs may benefit from explicit orientation that defines verbal abuse and clarifies that reporting is a safety behavior. A simple reporting algorithm (who to contact, what to document, and expected response time) may reduce uncertainty and lower barriers to early disclosure.
Adaptation phase (T2): Simulation-based de-escalation training, followed by structured debriefing or guided reflection, may help residents consolidate communication skills and normalize help-seeking.
Consolidation phase (T3): System-level reinforcement becomes increasingly important, including strengthening mentorship, closing feedback loops after reports, and ensuring transparent protection procedures. Reducing ambiguity about “who to tell and what happens next” may be essential for shifting verbal abuse from a silent burden to a manageable occupational risk (Elsharkawy et al., 2025).

### 4.6 Strengths and limitations

A key strength of this study is its zero-attrition longitudinal design, which captured within-person change across a critical transition year. The combination of IPA with a longitudinal matrix approach supported temporal comparison while preserving case-level interpretation.

Several limitations should be noted. First, the study was conducted in a single tertiary hospital, which may limit transferability to other settings. Second, accounts were retrospective at each wave and are therefore subject to recall bias; repeated interviews may also have influenced participants’ reflexivity. Third, the mandatory nature of the residency program likely contributed to the zero-attrition rate and may have shaped coping narratives under constrained conditions. Because all participants remained in the program, the findings may under-represent the most severe trajectories associated with early resignation, introducing potential survivorship bias. Future research should include multi-site sampling and extend beyond the first year to examine whether these trajectories remain stable over time.

Transferability should also be interpreted in relation to China’s sociocultural and historical context. Norms of endurance, hierarchical workplace relationships, and cohort-specific family and socialization experiences may shape how nurse residents understand resilience, authority, help-seeking, and professional maturity. Therefore, the coping trajectories identified in this study may not be directly transferable to countries or cohorts with different family structures, professional socialization patterns, or expectations regarding workplace conflict. Future multi-site and cross-cultural studies are needed to examine how these contextual factors influence appraisal and coping trajectories among newly graduated nurses.

## 5 Conclusion

Guided by Lazarus and Folkman’s transactional theory of stress and coping, this longitudinal qualitative study suggests that patient verbal abuse represents a persistent stressor for Chinese nurse residents, shaping their appraisal and coping trajectories over the first year of practice. Early accounts were characterized by definitional uncertainty, strong emotional reactions, and professional masking. Over time, many residents described more differentiated appraisals and more integrated coping approaches, alongside continued sensitivity to uneven institutional responses and unclear reporting pathways. These findings support the need for phase-matched interventions, including stage-appropriate communication and de-escalation training, clear and psychologically safe reporting mechanisms, and sustained peer and supervisory support, to facilitate professional identity development and strengthen the transition to practice.

## Data Availability

The original contributions presented in this study are included in the article/Supplementary material, further inquiries can be directed to the corresponding authors.
