# Supplementary material for: From silenced shock to strategic resilience: a longitudinal qualitative study of nurse residents’ trajectory in coping with patient verbal abuse
Source: Front Psychol. 2026 Jun 19;17:1780789. doi: 10.3389/fpsyg.2026.1780789 (PMC13327992; doi:10.3389/fpsyg.2026.1780789)
Supplement: Supplementary file 1 [file Supplementary_file_1.pdf]

Table S1. Semi-structured interview guide for the longitudinal study across three time points.

Interview Wave	Key Focus Areas (Derived from Lazarus & Folkman's Theory)	Sample Interview Questions and Probes
**Wave 1 (T1)**<br>(1 Month Post-Entry)	**Primary Appraisal:**<br>Initial shock; Defining the event.<br><br>**Emotional Response:**<br>Vulnerability; Self-blame.<br><br>**Early Coping:**<br>Immediate reactions; "Professional masking."	**1. The First Encounter:** Can you describe the most memorable incident of patient verbal abuse you have experienced since starting your residency? What specifically was said or done?<br>**2. Appraisal & Interpretation:** At that moment, what was your immediate thought? Did you interpret it as a personal failure or something else?<br>(Probe: Did you feel it was "abuse" or just the patient "venting"?)<br>**3. Emotional Impact:** How did you feel physically and emotionally during and after the incident? Did these feelings linger after your shift?<br>**4. Initial Coping:** How did you react in the moment? Did you speak up, stay silent, or leave the scene?<br>(Probe: You mentioned smiling while feeling terrible inside—can you tell me more about that experience?)<br>**5. Help-Seeking:** Did you tell anyone (preceptor, head nurse, or peers) about it? If not, what held you back?
**Wave 2 (T2)**<br>(6 Months Post-Entry)	**Re-Appraisal:**<br>Shifting perspectives; Cumulative stress.<br><br>**Coping Evolution:**<br>Peer support vs. hierarchy; Testing new strategies.	**1. Changes over Time:** Compared to your first month, how do you feel now when a patient raises their voice? Do you still feel the same level of shock?<br>**2. Coping Adjustments:** Have you tried any new methods to handle these situations proactively?<br>(Probe: Have you tried explaining procedures beforehand to prevent

	**Impact of "Masking":**<br>The "hidden exhaustion."	anger? Does it work?)<br>**3. The Cost of Silence:** We noticed many residents "hold it in." How is this suppressing of emotions affecting you now that six months have passed? Do you feel burned out?<br>**4. Social Support:** Who do you turn to now? Do you feel safer sharing with your fellow residents or your teachers?<br>(Probe: How does the "small group chat" with peers help you?)<br>**5. Institutional Response:** When you reported incidents to senior staff, how did they react? Did their reaction encourage you or silence you?
**Wave 3 (T3)**<br>(12 Months Post-Entry)	**Integration & Meaning:**<br>"Threat" vs. "Challenge"; Professional identity.<br><br>**Advanced Coping:**<br>De-escalation skills; Selective reporting.<br><br>**Recommendations:**<br>Needs for future training.	**1. Reframing:** Now that you are finishing your first year, how do you distinguish between a patient in pain and personal abuse? Does it still hurt your self-esteem?<br>**2. Strategic Response:** You seem calmer now. Can you describe your current step-by-step process when handling an aggressive patient?<br>(Probe: How do you balance "getting the work done" with "protecting your dignity"?)<br>**3. Selective Reporting:** We noticed you don't report every incident anymore. What is your criteria now for deciding when to report to a manager?<br>**4. Reflection on Growth:** Looking back, do you see these experiences as purely negative, or have they contributed to your professional growth?<br>**5. Institutional Advice:** If you could design a training course for the next group of nurse residents, what specifically should the hospital teach them about verbal abuse?

Table S2. Contextual characteristics across timepoints (N = 18).

A. Unit type at each interview wave

Unit type	T1 n (%)	T2 n (%)	T3 n (%)
Medical wards	4 (22.2)	3 (16.7)	4 (22.2)
Surgical wards	3 (16.7)	4 (22.2)	3 (16.7)
Emergency department (ED)	3 (16.7)	3 (16.7)	4 (22.2)
Intensive care unit (ICU)	3 (16.7)	2 (11.1)	2 (11.1)
Obstetrics/Gynecology (OB/GYN)	2 (11.1)	2 (11.1)	1 (5.6)
Pediatrics	2 (11.1)	2 (11.1)	2 (11.1)
Other	1 (5.6)	2 (11.1)	2 (11.1)
Total	18 (100.0)	18 (100.0)	18 (100.0)

B. Night shift exposure at each interview wave

Night shift exposure	T1 n (%)	T2 n (%)	T3 n (%)
Yes	14 (77.8)	16 (88.9)	17 (94.4)
No	4 (22.2)	2 (11.1)	1 (5.6)
Total	18 (100.0)	18 (100.0)	18 (100.0)

Note. Night shift exposure indicates whether the participant had worked ≥1 night shift during the current rotation prior to each interview wave (Yes/No).

C. Prior WPV-related training before T1

Prior WPV training	n (%)
Yes	0 (0.0)
No	18 (100.0)
Total	18 (100.0)

Note. Prior WPV-related training refers to any structured education or training (e.g., hospital orientation, in-service sessions, or school-based modules) on workplace violence prevention/management, de-escalation, or reporting completed before T1.

Table S3. Themes and subthemes across T1–T3 with exemplar quotations (N = 18).

Theme	Subtheme	Core meaning	T1 exemplar quote	T2 exemplar quote	T3 exemplar quote
1. Emotional shock and psychological vulnerability	1.1 Definitional ambiguity and early normalization	Difficulty distinguishing abuse vs venting; early normalization	P1, T1: "When the family member pointed at me and said 'How can you not know anything', my first reaction was to doubt myself. I kept wondering whether this counted as being bullied or whether he was just anxious. In the end I told no one and thought maybe work is just like this." P12, T1: "The patient used nasty words and I froze. I said I was fine, but inside I was deeply upset. I felt pain might make them like this, and reporting it would make me look petty or unable to endure."	—	—

1. Emotional shock and psychological vulnerability	1.2 First-encounter shock and emotional unpreparedness	Acute surprise; unpreparedness	P5, T1: "At school we learned empathy and caring. I was completely unprepared for someone to curse me like that. That whole day I was absent-minded and wondered whether I had chosen the wrong profession."<br>P7, T1: "I had imagined that patients might be dissatisfied, but I did not expect someone to speak to me with such harsh words. At that moment, I felt embarrassed and helpless, and I kept asking myself whether I was too inexperienced or too fragile. After work, I still could not calm down and wondered whether I was really ready to become a nurse."	—	—
1. Emotional shock and psychological vulnerability	1.3 Lingering impact of subtle remarks	Rumination; confidence erosion	P03, T1: "An elderly patient said, 'Why did they send a little rookie? Can she even figure it out?' I heard it clearly. That sentence stayed in my head and made me tense before every procedure."	P8, T2: "When someone says, 'Oh, another new one today?', I can smile it off, but it still weighs on me and makes me question whether I seem unprofessional."	—

1. Emotional shock and psychological vulnerability	1.4 Emerging regulation and reframing	More contextual appraisal	—	—	P15, T3: "Now I try to calm myself first and not be pulled into their emotions. I focus on what problem needs to be solved. If the patient is just anxious or uncomfortable, I explain and continue the care. But if the words become insulting or threatening, I know it should be documented or mentioned to someone I trust."<br>P17, T3: "Now I can tell the difference. If they lash out from pain or fear, I try to understand and not take

					it personally. I know it's not my failure. Pure personal attacks are another matter."
2. Struggling within hierarchical and cultural constraints	2.1 Professional masking and emotional suppression	Professional composure with internal strain	P2, T1: "I kept a smile on my face and said 'I understand', but my hands were shaking and my stomach was twisting. I couldn't cry or talk back, otherwise the situation would get worse and others would think I couldn't handle it."	—	—
2. Struggling within hierarchical and cultural constraints	2.2 Hierarchical constraints and fear of negative labeling	Avoiding escalation; stigma concerns	P9, T1: "I didn't dare tell the head nurse. She might think my communication caused it or that I was making trouble. New nurses fear being labeled as 'too sensitive'."	P14, T2: "A senior teacher sighed, 'If you can't even take this, what will you do later?' After that, I only vented with peers who understood."	—

2. Struggling within hierarchical and cultural constraints	2.3 Role stabilization via mentorship and experience	Mentorship reduces self-blame; strengthens belonging	—	—	P11,T3:"My preceptor shared similar experiences and taught me how to respond. More importantly, she made me feel it wasn't my fault. With her backing me up, I no longer felt like a lonely rookie."<br>Another resident similarly emphasized that mentorship helped transform the incident from a personal failure into a manageable workplace problem:<br>P10, T3: "My preceptor helped me review the situation instead of simply telling me to endure it. She said the patient's anger was not necessarily my fault and taught me how to explain, pause, and call for help if needed. After that, I felt more secure in the ward."

3. Gradual development of coping and adaptive strategies	3.1 Informal peer support as the safest early resource	Safest early emotional buffer	P04, T1: "After work my roommate and I cried and told each other, 'It's not our fault.' Realizing others had been through it reduced my shame and loneliness."	P16, T2: "We had a small group without teachers—our 'safe dump'. We shared what happened and how to respond, as a way to keep each other afloat."	—
3. Gradual development	3.2 Expansion into skill-based	Expectation management;	—	P7, T2: "Now I explain first: 'This needle may	—

of coping and adaptive strategies	coping and de-escalation	anticipatory explanation		hurt; I'll be as gentle as I can, but please keep still.' When I say it upfront, they complain less. It really works."	
3. Gradual development of coping and adaptive strategies	3.3 Integrated coping and selective institutional engagement	Emotion regulation + boundaries + selective documentation	—	—	P15, T3: "I stay calm and bring the focus back to the problem. Afterward I recover on my own. But if it feels threatening, I leave a note in the handover or mention it to a trusted leader—at least there should be a record."
4. Tension between professional	4.1 Early threats to professional	Early role insecurity	P6, T1: "After being yelled at 'Get out', I hid in the treatment room. My mind went blank. I thought maybe I	—	—

growth and institutional support	identity		really can't do thisjob."		
4. Tension between professional growth and institutional support	4.2 Uneven supervisory responses shaping help-seeking	Support vs minimization	—	Supportive—P10, T2: "My head nurse said first, 'You've been wronged.' She analyzed it with me and taught me what to do next time. I felt protected." Minimizing—P13, T2: "My preceptor just said 'mm-hmm' without looking up. It felt like cold water—like I was bothering her with something trivial."	—
4. Tension between	4.3 Demand for structured	Need for clear guidance and	—	—	P18, T3: "We got plenty of technical training, but no

professional growth and institutional support	training and clear pathways	predictable follow-up			one taught us how to respond to nasty words. I wish there had been clear guidance: who to go to, how to report, and what would happen next."
5.Cross-cutting negative case trajectory	Persistent threat-focused appraisal and constrained coping	Some residents did not show clear movement toward reframing or integrated coping. They continued to experience self-blame, avoidance, emotional suppression, and limited trust in reporting pathways.	P6, T1: "When I was scolded for the first time, I immediately wondered whether I had done something wrong or was simply not competent enough. I knew the patient might have been anxious, but I still felt that the words were directed at me personally. After the shift, I kept thinking that maybe I was not suitable for nursing."<br>P14, T1: "The first time a patient shouted and kept apologizing, but inside I felt embarrassed and angry. I also wondered other nurses seemed to handle it more calmly."	P6, T2: "After six months, I still usually chose to keep silent. I would finish the task as quickly as possible and then avoid staying with that patient for too long. I rarely reported these incidents, because I felt reporting might not change anything and might make others think I was too sensitive or troublesome."<br>P14, T2: "Now I look calmer on the outside, but I do not think I have really adapted. I just know better how to hide my feelings. When something like this happens, I usually	P6, T3: "Even now, when a patient scolds me, my first reaction is still to wonder whether I did something wrong. I know I have worked for almost a year, but I still cannot completely separate their words from my own ability."<br>P14,T3:"I rarely report these incidents, not because they no longer affect me, but because I do not think reporting will change anything. Sometimes I also worry that leaders will think I am too sensitive or troublesome."

				keep quiet and tell myself to get through the shift first.”	

Note. Exemplar quotations were purposively selected to illustrate each subtheme and reflect variation across participants and timepoints; the table is illustrative rather than exhaustive. Quotations are presented in English translation. T1 ≈ 1 month, T2 ≈ 6 months, and T3 ≈ 12 months after entering clinical practice.
